# Security Issues in Healthcare Applications Using Wireless Medical Sensor Networks: A Survey

**DOI:** 10.3390/s120100055

**Published:** 2011-12-22

**Authors:** Pardeep Kumar, Hoon-Jae Lee

**Affiliations:** Department of Ubiquitous-IT, Graduate School of Design & IT, Dongseo University, San 69-1, Jurye-2-Dong, Sasang-Gu, Busan 617-716, Korea; E-Mail: pradeepkhl@gmail.com

**Keywords:** healthcare applications, healthcare security issues, patient privacy issues, medical sensor networks, wireless sensor network, wireless body area network

## Abstract

Healthcare applications are considered as promising fields for wireless sensor networks, where patients can be monitored using wireless medical sensor networks (WMSNs). Current WMSN healthcare research trends focus on patient reliable communication, patient mobility, and energy-efficient routing, as a few examples. However, deploying new technologies in healthcare applications without considering security makes patient privacy vulnerable. Moreover, the physiological data of an individual are highly sensitive. Therefore, security is a paramount requirement of healthcare applications, especially in the case of patient privacy, if the patient has an embarrassing disease. This paper discusses the security and privacy issues in healthcare application using WMSNs. We highlight some popular healthcare projects using wireless medical sensor networks, and discuss their security. Our aim is to instigate discussion on these critical issues since the success of healthcare application depends directly on patient security and privacy, for ethic as well as legal reasons. In addition, we discuss the issues with existing security mechanisms, and sketch out the important security requirements for such applications. In addition, the paper reviews existing schemes that have been recently proposed to provide security solutions in wireless healthcare scenarios. Finally, the paper ends up with a summary of open security research issues that need to be explored for future healthcare applications using WMSNs.

## 1. Introduction

Wireless sensor networks (WSNs) are an emerging technology in existing research and have the potential to transform the way of human life (*i.e.*, make life more comfortable). A wireless sensor is the smallest unit of a network that has unique features, such as, it supports large scale deployment, mobility, reliability, *etc.* WSNs are not limited to science and engineering, but they are also included in other popular applications such as the military, water monitoring, infrastructure monitoring, government security policy, habitat monitoring, environment monitoring, and earthquake monitoring, are few examples. A sensor network consists of a discrete group of independent nodes with low cost, low power, less memory, and limited computational power that communicate wirelessly over limited frequencies at low bandwidth [1]. The main goals of WSNs are to deploy a number of sensor devices over an unattended area, and collect the environmental data and transmit it to the base station or remote location. Later, the raw data is processed online or offline for detailed analysis at the remote server according to the application requirements.

### 1.1. Background

**Figure 1.**
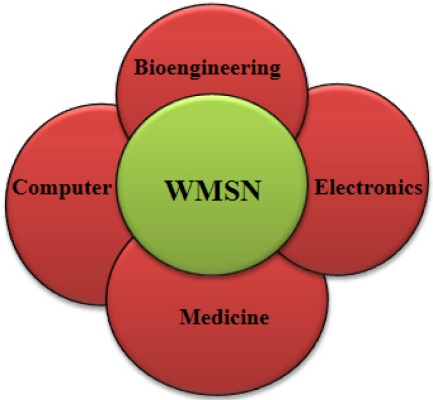
Interdisciplinary research of WMSN.

In the 21st century, the healthcare industry has seen the drastic improvements due to the involvement of wireless medical sensor networks (WMSNs) in healthcare applications. A few decades ago WSNs were a topic of science/movie fiction for healthcare industries, and now they have become a reality and provide much quality-of-care. As the world’s aging population is increasing at an unprecedented rate in the developed and developing countries. According to the “An Aging World: 2008” report [2], in 2008 the number of aging people worldwide (*i.e.*, 65 years and older) was estimated at 506 million, and by 2040, that number will touch 1.3 billion. Thus, in just over three decades, the percentage of older age people will increase two times from 7% to 14% of the total world population [2]. Although the aging population signifies, a human success story of increased longevity, the steady, sustained growth of the older population also poses health challenges. As more and more people will be entering an elder age, the risk of developing certain chronic and debilitating diseases is significantly higher. For example, Alzheimer disease symptoms typically first appear after age 60 [3], Heart disease and stroke rates rise after age 65 [4], diabetes, like those of many other conditions (e.g., blood pressure, blood glucose levels *etc.*). Further, if aged populations prefer to live alone they do however require long-term monitoring for better independent life [5]. Thus the aging population desperately demands independent life and good quality-of-care without disturbing their comfort, while reducing their care costs. In this context, wireless sensor technology could provide highly useful tools for elderly people health monitoring and patients who need continuous monitoring. Consequently healthcare using wireless sensor networks constitutes an exciting and growing field for scientific investigation. In fact the future of modern healthcare in an aging world will need ubiquitous monitoring of health with least actual interaction of doctor and patients [6]. Recently, a term wireless medical sensor network (WMSN) has coined to bring many researchers together from interdisciplinary areas (bioengineering, electronics, computer, medicine), as shown in Figure 1.

Wireless medical sensors may be wearable, implantable or portable, and integrated on various kinds of wireless communication motes (such as, Mica2, MicaZ, Telos, *etc.*). A typical Mica2 mote has a 7.3 MHz Atmel ATmega128L CPU with 128 KB of ROM, and 4 KB of RAM for data [7]. The radio operates at 76.8 Kbps bandwidth at a range of a few meters. Moreover, a sensor node typically has a limited battery power (e.g., AA-batteries), which is just enough for communication (e.g., unicast, multicast and broadcast) and computation [7]. Furthermore, WMSNs are different from generic WSNs. The main differences are summarized in Table 1.

As we can see from the Table 1, generic WSNs are automatic and standalone, deployed at a large scale in either a fixed or distributed manner, and their data rates depend specifically on the applications, whereas WMSNs have direct human involvement (*i.e.*, patient, doctor, nurse, *etc.*), are deployed at a small scale (*i.e.*, depending on usability), must support mobility (a patient can carry the devices), and WMSNs requires high data rates (e.g., ECG data is normally sampled at a rate of 250 Hz and blood pressure at 100 Hz [8]), with reliable communication and multiple recipients [9]. Wireless medical sensor motes are deployed on a patient’s body, and are used to closely monitor the physiological condition of patients. These medical sensors sense the patient’s vital body signs and transmit the sensed data in a timely fashion to some remote location without human intervention. A doctor can use these medical sensor readings and gain a broader assessment of a patient’s health status. The patient’s vital signs may include heart beats, temperature, blood pressure, motion/acceleration, pulse-oximetry *etc.* Thus patients could benefit for continuous long-term monitoring after returning home from the hospital.

**Figure 2.**
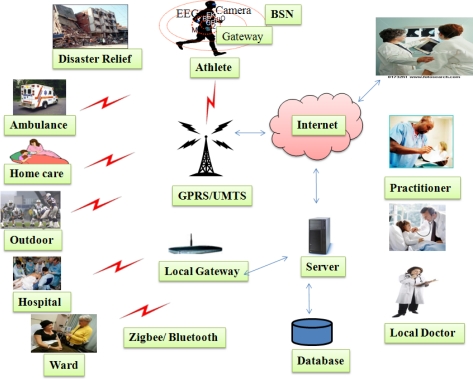
Healthcare application using wireless medical sensor networks.

As shown in Figure 2, WMSNs carry the promise of quality-of-care across wide variety of healthcare applications (e.g., ambulatory monitoring, vital sign monitoring in-hospitals, elderly peoples’ at home care monitoring, monitoring in mass-casualty disasters, clinical monitoring, *etc.*).

In addition, other applications that also benefit from WMSNs include sports-person health status monitoring [10], and patients’ self-care (*i.e.*, a BAN network on a diabetic patient could be helpful to auto inject insulin though a pump, as soon as their insulin level declines).

So far several research groups and projects have started to develop health monitoring using wireless sensor networks, for example, CodeBlue [7], LiveNet [11], MobiHealth [12], Alarm-Net [13], UbiMon [14], ReMoteCare [15], MobiCare [16,17], Lifeguard [18], AID-N [19], CareNet [20], ASNET [21], WiMoCa [22], SAPHIRE [23], THE-MUSS [24]. Thus, healthcare systems are the most beneficial applications using wireless medical sensor technology that can perform patient care within homes, hospitals, clinics, disaster sites and the open environment.

### 1.2. Problem Statement

**Figure 3.**
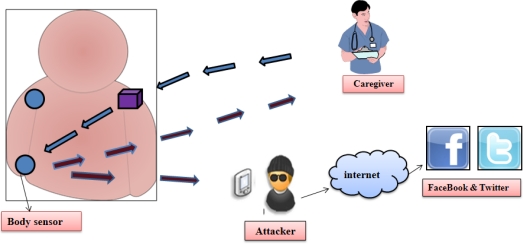
Risks to patient privacy.

The development of a wireless healthcare application offers many novel challenges, such as, reliable data transmission, node mobility support and fast event detection, timely delivery of data, power management, node computation and middleware [25–32]. Further however, deploying new technologies in healthcare applications without considering security often makes patient privacy vulnerable [8,33–36]. For instance, the patient’s physiological vital signals are very *sensitive* (*i.e.*, if a patient has some embarrassing disease), so any leakage of individual disease data could makes him/her embarrassed. In fact sometimes revealing disease information can result in a person losing his/her job, or make it impossible for him/her to obtain insurance protection [37]. Further, wireless medical sensor networks cover a broad range of healthcare applications, such as physiological data monitoring, and activity monitoring in health-clubs, location tracking for athlete, *etc*. Consequently, WMSNs share individual data with physicians (in a doctor-patient relationship), insurance companies (as insurance protection), and health-coaches (as sports team trainers) or with family (as relatives’ support) [38]. Therefore unauthorized collection and use of patient data by potential adversaries (such as insurance agents, for political reasons, rival coaches, personal enemies *etc.*) can cause life-threatening risks to the patient, or make the patient’s private matters publically available [37]. For example, in a simple scenario, a patient’s body sensors transmit his/her body data to a nurse/caregiver; it may happen that an attacker is also eavesdropping the patient data while the data is transmitting, and consequently the patient’s privacy is breached. Later that attacker can post the patient data on s social site (FaceBook or Twitter, *etc.*), and thus pose risks to the patient’s privacy, as depicted the Figure 3. Indeed wireless healthcare can offer many advantages to patient monitoring, but the physiological data of an individual are highly vulnerable, so security and privacy become some of the big concerns for healthcare applications, especially when it comes to adopting wireless technology. More importantly, a healthcare provider is subjected to strict civil and criminal penalties (*i.e.*, either fine or imprisonment) if HIPAA rules [39] are not followed properly. Thus a patient security and privacy is the central concern in healthcare applications.

Moreover, traditional security mechanisms needed unlimited resources, so they cannot be directly applied to the extremely resource-constrained sensor nodes. While WMSNs’ security requirements are the same as those of traditional networks, namely availability, confidentiality, integrity, authentication, data freshness and non-repudiations, thus resource conscious security protocols have emerged as one of the critical issue in healthcare applications using wireless medical sensor networks. There are other survey studies on security and privacy issues in wireless healthcare applications [8,33–36,40–43]. However, these studies discuss limited information about these security issues, so we can conclude that the topic of security in wireless medical sensor networks has not been properly investigated yet, which provides ample avenues to explore secure wireless healthcare applications.

### 1.3. This Paper’s Contribution

The contributions of this paper are as follows: we present the state of the art in WMSN healthcare projects that have been introduced over the last decade and discuss their security flaws. We broadly explore the possible security threats that can endanger healthcare applications, including patient’s privacy issues. Further this paper presents an overview of stringent government rules and regulations for healthcare organizations and their business partners. We also instigate discussion on existing security mechanisms that need to be explored, and sketch out the imperative security requirements in wireless healthcare. This paper also includes a holistic overview on recent available literature that has proposed solutions for secure healthcare application using WSNs.

The rest of the survey is organized as follows: Section 2 gives an overview of recent WMSN healthcare projects. Sections 3 discuss the security and privacy issues for wireless healthcare applications and Section 4 discusses the stringent regulations for healthcare organizations. Section 5 discusses the issues of existing security mechanisms, and Section 6 points out the inherent security requirements to develop a secure healthcare application. Section 7 presents the related works for secure healthcare applications using WMSNs. Section 8 provides discussions on several open issues that are required for the success of future healthcare applications and in Section 9 conclusions are drawn for wireless healthcare applications.

## 2. Healthcare Projects Using Wireless Medical Sensor Networks

The advancement of WMSNs in healthcare applications have made patient monitoring more feasible. Recently, several wireless healthcare researches and projects have been proposed, which aim to provide continuous patient monitoring, in-ambulatory, in-hospital, in-clinic, and open environment monitoring (e.g., athlete health monitoring). This section describes few of the popular research projects about healthcare systems using medical sensor networks.

CodeBlue [7,44] is a popular healthcare research project based on a medical sensor network developed at the Harvard Sensor Network Lab. In this architecture, several medical sensors (e.g., pulse oximeter, EMG, EKG, and SpO2 sensor board onto the Mica2 motes [45]) are placed on the patient’s body. These medical sensors sense the patient body data and transmit it wirelessly to the end-user devices (PDAs, laptops, and personal computers) for further analysis. The basic idea of CodeBlue is straightforward, a doctor or medical professional issues a query for patient health data using their personal digital assistant (PDA), which is based on a *publish* and *subscribe* architecture. The medical sensors *publish* their relevant data to a specific channel and end-user *subscribes* the channel by using their hand-held devices (e.g., PDA and laptop). A TinyADMR routing component is used that is based on an adaptive demand-driven multicast routing (ADMR) protocol. TinyADMR facilitates node mobility, multicast routing and minimal path losses. Further, the CodeBlue architecture facilitates RF-based localization (*i.e.*, called MoteTrack [46]), which is accurate enough to locate a patient’s or medical professional’s position. More importantly, CodeBlue’s authors acknowledge the need of security in medical applications, but until now security is still pending or they intentionally left the security aspects for future work, although, in [44] the authors suggested that elliptic curve cryptography (ECC) [47] is a good candidate for the key generation, and TinySec [48] is good for symmetric encryption in CodeBlue project. Further, Kambourakis *et al.* [49] have sketched-out some security threat and attacks on the CodeBlue project such as denial-of-service attacks, snooping attacks, modification attacks, routing loop attack, grey-hole attack, Sybil attack and masquerading attacks. They address suitable countermeasures for CodeBlue security; for details reader may refer to [49]. CodeBlue is anticipated for deployment in pre-hospital and in-hospital emergency care, stroke patient rehabilitation and disaster response. We assumed that the authors have left security work for future.

**Figure 4.**
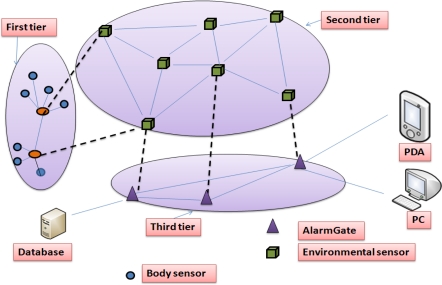
ALARM-NET architecture.

A heterogeneous network architecture named Alarm-Net was designed at the University of Virginia [13]. The research is specifically designed for patient health monitoring in the assisted-living and home environment. Alarm-net consists of body sensor networks and environmental sensor networks. Three network tiers are applied to the proposed assisted-living and home environment, as shown in Figure 4. In the first tier a resident wears body sensor devices such as ECG, accelerometer, SpO2 (*i.e.*, a MicaZ boards [50]) which sense individual physiological data; and in the second tier environmental sensors such as temperature, dust, motion, light (*i.e.*, MicaZ boards) are deployed in the living space to sense the environmental conditions. In the third tier an internet protocol (IP)-based network is used which is comprised of Stargate gateways called AlarmGate. The idea of Alarm-net is very simple, body sensors broadcast individual physiological data using single-hop to the nearest stationary sensor (*i.e.*, second tier). Thereafter, the stationary emplaced sensor nodes forward the body data using multi-hop communication (*i.e.*, shortest-path-first routing protocol) to the AlramGate. The AlarmGate is a gateway between the wireless sensor and IP networks, and is also connected to a back-end server.

Any real-time data queries about physiological or environmental data are originated by the user that contains the source address, ID, and sensor type. For a single-shot query, the sensors sample the requested data and respond a single report to the query originator, and hence complete the query. In addition, authors have developed a circadian activity rhythms program to aid context-aware power management and privacy policies.

Further Alarm-Net facilitates network and data security for physiological, environmental, behavioral parameters about the residents. Only authenticated users can access the Alarm-Net and can query the sensor networks. The IP-network is secured by secure remote password (SRP) protocol for user authentication. The wireless sensor networks are enabled with Link-layer security suites. Sensors (*i.e.*, MicaZ [50] and Telos [51]) use built-in cryptosystems, *i.e.*, an advanced encryption system (AES) for cryptographic operations. AES security modes supported are: none, CBC-MAC authentication-only, CTR mode encryption-only, and CMM combines with authentication and encryption [13]. The major drawback of the built-in cryptosystem is that it does not offer AES-based decryption, by which means the encrypted data cannot be accessed by an intermediary node during communication, if needed. Further, hardware based built-in cryptosystem makes the application highly platform dependent. More notably, Pai *et al.* have pointed out some confidentiality infringement scenarios on Alarm-NET, such as the fact it is susceptible to adversarial confidentiality attacks, which can leak resident’s location; refer to [52] for details.

UbiMon (Ubiquitous monitoring environment for wearable and implantable sensors) [14] is a BSN (Body Sensor Network) architecture composed of wearable and implantable sensors using an *ad hoc* network. The aim of the project is to provide continuous monitoring of an individual’s physiological states and capture transient as well as life threatening abnormalities that can be detected and predicted.

**Figure 5.**
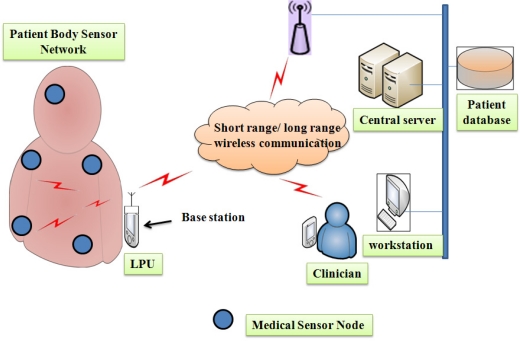
UbiMon system architecture.

As shown in the Figure 5, the UbiMon architecture consists of the following: (i) *BSN node*: Each node is integrated with bio-sensors (ECG, SpO2, temperature). (ii) *LPU (Local Processing Unit)*: LPUs can be portable devices (PDAs, laptop, *etc.*) used to the gather data from BSNs, and are known as the base station. They detect the patient’s abnormalities and provide immediate warning to the physician. Apart from this function, the LPU works as a router between BSN nodes and the central server using wireless communication (for short range Bluetooth/Wi-Fi and long-range mobile networks such as 3G/GPRS). (iii) *CS (Central Sever)*: A CS feeds the patient data to the PD (Patient Database), and can analyze the patient’s data on the basis of patient’s condition, and detect potential life-threatening abnormalities. (iv) *WS (Work-station)*: The WS is the patient’s data monitoring terminal (PC/laptop), which is used by the physician. Although Ng *et al.* proposed and demonstrated the ubiquitous healthcare monitoring architecture, it is widely accepted that without considering security for such applications they are often vulnerable to security attacks. So the authors did not consider security for wireless healthcare monitoring, which is a paramount requirement of healthcare applications, according to government laws [39].

**Figure 6.**
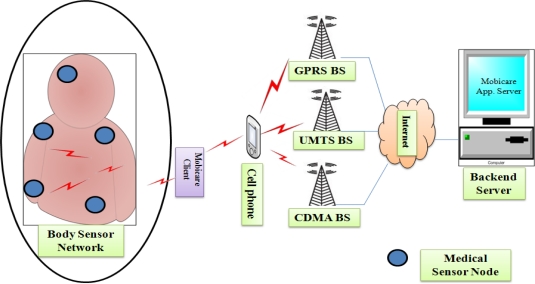
MobiCare patient monitoring architecture.

In 2006, Chakravorty designed a mobile healthcare project called MobiCare [16]. MobiCare provides a wide-area mobile patient monitoring system that facilitates continuous and timely monitoring of patients physiological status. It (MobiCare) potentially improves the quality-of-patient care and saving many lives. As shown in the Figure 6, the proposed system comprises of body sensor network (BSN) having wearable sensors (e.g., ECG, SpO2, and blood oxygen); a BSN manager called “MobiCare client that is an IBM wristwatch”; and a back-end infrastructure (*i.e.*, MobiCare server). The medical sensors timely sense the patient’s body data and broadcast it to the MobiCare client. The MobiCare client aggregates the body data and sends them using GPRS/UMTS or CDMA cellular link to the MobiCare server. In this research, MobiCare client makes use of application layer standard HTTP POST protocol for sending BSN data to the server. The MobiCare server supports to the medical staffs for offline physiological analysis, and for patient care [16].

Although, Chakravorty acknowledged the security issues in MobiCare, but only addressing security issues are not sufficient for real-time healthcare applications. In fact author suggested that the wireless application protocol (WAP), which is based on wireless transport layer security (WTLS) protocol could be used to provide the patient privacy, data integrity and authentication. Thus, security and privacy is still not implemented in MobiCare healthcare monitoring, or may have been left out for future work.

In [53] authors proposed a personal ambient monitoring (PAM) project for mental health monitoring. The project aims to monitor the activity signatures of patients with bipolar disorder (BP). The PAM has two levels, namely, a personal ambient monitoring infrastructure (PAM-I) and a personal ambient monitoring programming architecture (PAM-A). PAM-I is composed of body and environmental sensors, mobile phones, and personal computers. The medical sensors are planted on an individual’s body and environmental sensors are placed in the home environment. The Bluetooth protocol used to connect the body sensors and mobile phones; further Bluetooth also connects the mobile phones and personal computers. Mobile phones are responsible for data aggregation from body sensors and send it to the PC for storage and analysis. It (the mobile phone) controls sensor collection, *i.e.*, mental illness, using rule-oriented applications. The environmental sensors transmits data to the PC using multiple communication protocols (*i.e.*, IEEE 802.11 b/g, X10, Bluetooth, *etc.*) [53]. On the other hand PAM-A is composed of custom applications that handle inter-device network communication (*i.e.*, control and record the data, and transfer data offsite for analysis). PAM-A supports the mobile phone and the PC, further PAM-A applications are written in Java and Prolog (*i.e.*, rule-based programming). This is the first attempt to obtain activity signatures from the mentally ill patient using worn and environmental sensors networks. Although, authors blaze a new research trail for mental illness monitoring, they focused mainly on reliability and acceptability issues. Although the authors are mainly concerned about wireless mental health monitoring applications where patient privacy is an imperative requirement for such applications, they did not address patient privacy, which is unacceptable for such healthcare applications.

**Figure 7.**
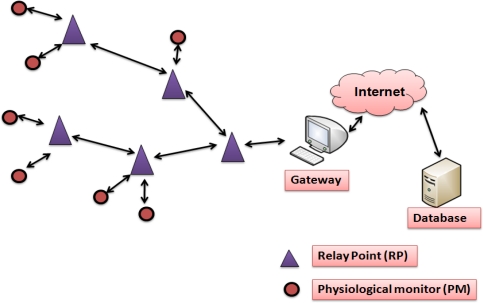
Healthcare architecture of MEDiSN.

Recently, a new system designed at Johns Hopkins University named MEDiSN, especially designed for patients’ monitoring in hospital and during disaster events was reported [54]. It comprises multiple physiological monitors (called PMs), which are battery powered motes (*i.e.*, Telos boards [51]) and equipped with medical sensors for collecting patients’ physiological health information’s (e.g., blood oxygenation, pulse rate, electrocardiogram signals, *etc.*), as shown in Figure 7.

The PMs are mobile, temporarily storing sensed data and transmitting it (after encrypting and signing the sensed data) to the relay points (RPs). MEDiSN incorporates different stationary RPs that are self-organized into a bidirectional routing tree, and forwards PM data to the gateways and *vice versa*. The RPs uses a collection tree routing protocol (CTP) to forward their measurements to the gateway. Moreover, MEDiSN is connected with a back-end database that constantly stores medical data and presents them to authenticated GUI clients. Specifically, this research focused on reliable communication, data rate, routing, and QoS [54].

In their description of MEDiSN its authors acknowledged the need for encryption for PMs, but they did not describe which cryptosystem has been used for data confidentiality and how they have checked the authenticity of the delivered data. Thus, although the authors provided security to MEDiSN, their study did not reveal much information about their security implementation. Further, only authenticated clients can access and control the sensor network at back-end server, but which authenticated protocol they have used is unknown. As a result, from a security perspective authors failed to provide detailed information about their security mechanisms.

In [55], the authors designed and developed a wearable personal monitoring service, called SATIRE, with the collaboration of the University of Illinois and the University of Virginia. SATIRE allows users to maintain a private searchable record of their daily routine activities (e.g., measured by two motion and location sensors). A person wearing a SATIRE jacket that can record his/her normal daily activities. When the person (*i.e.*, wearing a jacket) comes into the vicinity of an access mote (*i.e.*, connected to a personal computer), the logged data is uploaded reliably to a private repository associated with that person. Later, this data may be used to reconstruct the activities and locations of the person. Although, authors properly address security and privacy issues in SATIRE, they did not implement any security and privacy for sensitive physiological data. We must assume that the authors intentionally considered security a topic for future work.

As we have seen, all the above on-going healthcare monitoring projects enable automatic patient monitoring and provide potentiated quality of healthcare without disturbing patient comfort. All the projects focus on the reliability, cost effectiveness and power consumptions of their prototypes, but although most of the healthcare projects mentioned above addresses the need for security and privacy for sensitive data (e.g., CodeBlue [7,44], MobiCare [16], STAIRE [55]), only a few embed any security (e.g., ALARM-NET [13], MEDiSN [54]), which is not sufficient for such critical applications. Hence, security and privacy have not been investigated in much depth, and challenges still remain for real-time wireless healthcare applications.

## 3. Security and Privacy Issues

**Figure 8.**
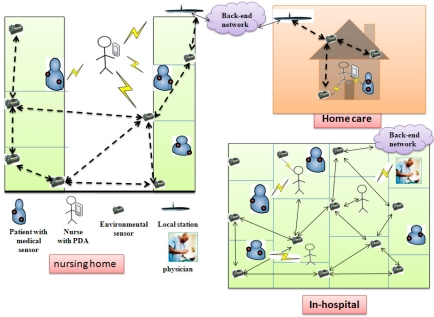
Application scenarios for a nursing home, home care, and in-hospital.

This section discusses: (i) which would be the possible threats to a wireless healthcare application without implementation of proper security; and (ii) privacy issues. Before discussing the security issues in wireless healthcare applications, it is worthwhile to assume the scale of deployment of healthcare applications using WMSNs. In this regards, we have considered three wireless healthcare scenarios, namely, a nursing home, in-home monitoring, and in-hospital monitoring, as shown in Figure 8.

The wireless healthcare applications use medical sensors (*i.e.*, as *per* patient appropriateness) and environmental sensors (ES), mobile devices (*i.e.*, PDA, laptop or iPhone), and more especially wireless communication (*i.e.*, IEEE 802.11, IEEE 802.15.4, Bluetooth *etc*.) protocols. Further, a back-end server is used for physiological healthcare information (PHI) storage, and for offline analysis of PHIs. According to the *nursing home scenario* Figure 8 (left), medical sensors are placed on a patient’s body and sense the physiological data of an individual and transmit it in a timely way to the PDA that may be held by a nurse. A nurse can query the patient’s sensors and analyze the real-time patient data conditions. Later she can send patient data to the central server either using Internet or a wireless medium. Many ES are deployed in nursing homes that can form a wired or wireless network, sense the environmental parameters (e.g., ward temperature, humidity, *etc.*) and transmit the data to either a nurse or a remote center. In addition, the environmental sensors may forward an alarm to the remote server in an emergency situation (e.g., supposing a severe condition is detected), should one occur. In the *home scenario* (Figure 8, upper right), medical sensors are planted on a patient’s body and capture the health data from an individual and transmit it in a timely fashion to a PDA held by a nurse or family member. In addition, environmental sensors are required when a patient is usually alone at home. The environmental sensors are placed at the corners of rooms, collecting the environmental conditions (e.g., room temperature, humidity, *etc.*), and patient movement data. Later they automatically send collected environmental and patient abnormal conditions to the PDA, which is held by either a nurse or a responsible family member. The home local station can directly communicate with environmental sensors using Zigbee modules. To analyze the patient physiological data an application program will be implemented at the back-end network. In the *In-hospital scenario* (Figure 8, bottom right), the same deployment and sensing scenario (*i.e.*, as in the nursing home and homecare scenarios) is now applicable to the hospital environment, where groups of patients are temporarily monitored using a wireless medical sensor network by nurses or physicians using their PDAs; for more details the reader may refer to [56].

### 3.1. Security Threats

As we have seen in the above healthcare scenarios, WMSNs certainly improve patient’s quality-of-care without disturbing their comfort. The medical sensor senses patient *sensitive* body data and transmits it over the wireless channels which are more susceptible than wired networks. Thus, patient sensitive physiological variables must remain secure and private from security threats, so this sub-section discusses the possible security threats that would be harmful for the wireless healthcare success, as follows:

#### Monitoring and Eavesdropping on Patient Vital Signs

This is the most common threat to the patient privacy. By patient vital sign snooping, an adversary can easily discover the patient information from communication channels. Moreover, if the adversary has a powerful receiver antenna, then he/she can easily pick up the messages from the network. The captured message may contain the physical location of the patient, allowing an attacker to locate the patient’s position and physically harm him/her. In addition, an adversary can also detect the message contents including message-ID, timestamps, source address, destination address and other relevant information. Thus, monitoring and eavesdropping can pose a serious threat to patient privacy [8,16].

#### Threats to Information When in Transit

As we know, wireless communication ranges are not confined, and are easily vulnerable. In wireless healthcare applications, medical sensors sense the patient and environmental data, and send it either to the physician or the hospital server. While sending the sensor’s data (*i.e.*, in transit), it may be attacked. For example, an adversary can capture the physiological data from the wireless channels, and can alter the physiological data. Later, he/she may pass the attacked data (*i.e.*, altered data) to the physician or remote server, which could endanger the patient. There are various types of in transit attacks: (i) *Interception:* Suppose, a WMSN has been compromised by a smart adversary. Then he/she can illegally access the sensor node data (e.g., cryptographic keys, sensor ID’s, type *etc.*). (ii) *Message modification*: In the message modification attack, the attacker can capture the patient wireless channels and extract the patient medical data; and later he/she may tamper with the patient data, which can mislead the involved users (e.g., doctor, nurse, family member, *etc.*). For example, suppose a cardiograph sensor transmits normal data to the medical staff, if an attacker able to modify the patient data during the communication and send the modified data to medical staff, it may cause an overdose of medicine being administered to the patient. Further, this modified data can trigger a false alarm or can hide the true patient conditions, if abnormal. Message modification threatens the message integrity of medical sensor nodes.

#### Routing Threats in WSNs

**Figure 9.**
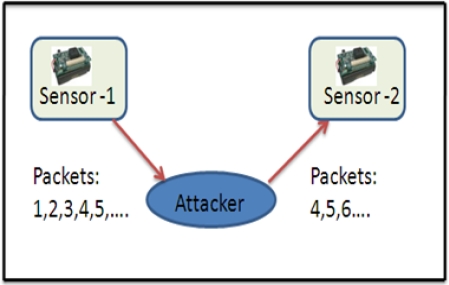
Selective Forwarding Attack.

**Figure 10.**
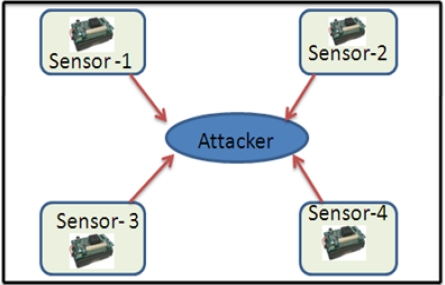
Sinkhole Attack.

**Figure 11.**
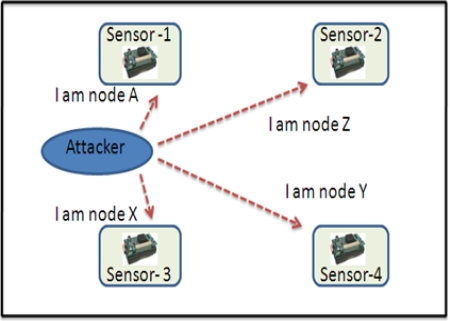
Sybil Attack.

For the experimental scenario, consider the CodeBlue [7] and MEDiSN [54] and [57] architectures, which need a multi-hop environment (*i.e.*, one node to another node) from body sensors to a remote server. A malevolent user could thus attack the network layer. He/she may steal or modify the packets, and forward the altered packets to the remote center (*i.e.*, back-end) that may cause a false alarm. More specifically in the CodeBlue [7] application, body sensors send their data using an ADMR routing protocol to the remote location (*i.e.*, hospital). An attacker might alter the address fields of captured packets before forwarding them to the next hop, and consequently, misguide the route or even generate an endless routing loop [49]. The routing attacks in a multi-hop environment are the following:

- Selective forwarding: In multi-hop environment [7,54,56,57], sensor packets (*i.e.*, health data or environmental data) are expected to be forwarded to the base station or remote server via multi-hop routing. In this threat, malicious nodes may refuse to forward certain messages (e.g., ECG, temperature, *etc.*) and may simply drop them, so that they cannot be broadcast further. This threat can be stronger if the attacker is explicitly included in the routing path. Figure 9 illustrates an example; suppose an ECG sensor forwards packets, *i.e.*, 1, 2, 3, 4… 10, to the next hop. But if a patient’s enemy intentionally capture and drops some ECG packets, and only forwards a few packets, *i.e.*, 4, 5, 6, 8 and 10, to the remote site [58], this could be life-threatening in a patient emergency condition. Further, in [49], the authors point-out that the CodeBlue architecture is susceptible to grey-hole attacks. They claimed that if attackers modify the ADMR packet header of certain packets by small hop-count, they can make the adjacent nodes believe that the attacker is located in the shortest-path to the sink. Thereafter, the attacker can generously drop every packet, whatever he/she receives. That could cause life-threatening risks.
- Sinkhole threat: In this threat, an attacker tries to attract all neighboring nodes to establish routes through a malicious node. Figure 10 illustrates a sinkhole attack; once the attacker is successful in the sinkhole attack, then the network is also open to other attacks [58], for example, eavesdropping or selective forwarding. Sinkhole attacks are very hard to detect [59].
- Sybil Attack: In this attack, a compromised node presents multiple fake identities to other neighboring nodes in the network [60]. Figure 11 illustrates how the multiple fake identities of a compromised node are sent to other neighboring nodes [58]. Further, Kambourakis *et al.* claim that the CodeBlue system is susceptible to Sybil attack, especially, when it operates in an *ad-hoc* manner; for details the reader can refer to [49]. The Sybil attack poses a significant threat in geographic and multipath routing protocols, because the compromised node may appear in more than one place [61]. Further, in [60,62] the authors have discussed more routing attacks in the context of healthcare applications using sensor networks, e.g., sleep attack, fairness attack, and wormhole attack.

#### Masquerade and Replay Threats

In a home care application, an attacker can easily rogue a wireless rely point while patient data is transmitting to the remote location. In general wireless rely nodes are unguarded, so it may happen that a rogue rely node can provide unrestricted access to an attacker who can then cause a masquerade. In this threat, an illegal rely node acts as a real node to the network. This can lead to false alarms to remote sites and an emergency team could start a rescue operation for a non-existent person. A masquerade node can apply easily denial-of-service attacks, and can disrupt the application operation. It can even defeat the purpose of wireless healthcare. Thus, masquerading nodes can be very dangerous for healthcare applications. More important, if a masquerade relay node captures the patient physiological data, later, these captured messages can pose replay threats to the real-time healthcare application. Obviously the patient treatment depends on fresh received messages from medical sensor networks. If masquerade nodes replay the old messages again and again, this could cause of mistreatment and overtreatment (*i.e.*, medicine overdose) of the patients. Thus, masquerade and replay threats endanger real-time healthcare applications using wireless medical sensors.

#### Location Threats

Medical sensor networks support patient mobility, so exact patient location knowledge is needed since location knowledge allows reaching medical staff in a short time, in case of any emergency [63]. Generally, location-tracking systems are based on radio frequency [46], ultrasound, received signal strength indicator or some other technology [44]. Curtis *et al.* [64] have used geo-positioning to locate the patient and caregiver in their project called SMART (Scalable Medical Alert Response Technology). As the authors support localization system for the patients and medical staff, if an adversary constantly receives the persons’ radio signals and analyze them, then he/she gains details of those persons’ locations, which could directly infringe a person’s privacy.

#### Activity Tracking Threats

An adversary can obtain someone’s health status while he/she is busy exercising in a health-club. Based on a sensor’s captured data a malevolent user can guess the current activity of a patient and he may send the wrong exercise tips to the patient that could cause them severe pain. Considering another example, an athlete is being monitored using a wireless sensor network while he/she is practicing in the club [65]. Medical sensors are placed on the athlete’s body, which sense health data, e.g., heart-rate, time and location, and send health feed back to the base station [10], so it might be possible for an adversary to modify the athlete’s health data, which may bring the athlete under suspicion in doping tests that could even spoil the athlete’s career.

#### Denial-of-Service (DoS) Threats

In [66] Wood-Stankovic stated that “a denial-of-service attack is any event that diminishes or eliminates a network’s capacity to perform its expected function.” Denial-of-service threat could be even more disruptive in healthcare applications because such a network needs always-on patient health monitoring (*i.e.*, in-home, in-hospital, ambulatory *etc*.). As DoS threats are harmful to every application, we have directly adopted DoS from [66]. A list of denial-of-service attacks is shown in Table 2.

- Physical layer: Jamming and tampering are the most common attacks on the physical layer. In jamming, generally, an attacker can squeeze the communication using high radio frequency (RF) signals, which disturb the network functionality. For example, medical sensor networks are small networks that squeeze early. Tampering is also known as a type of physical attack. A malevolent user steals the medical sensor and electronically interrogates it to extract the patient information from the sensor mote.
- Link/medium access control layer: This layer suffers mainly from collision, exhaustion, and unfairness attacks. In collision attacks, an adversary simultaneously transmits the packets at same frequency, resulting in packet collision and degradation of the network performance. In exhaustion attacks the battery source is self-sacrificing, since wireless nodes most of the time maintains the channel active. In unfairness attacks, network performance degrades because medium access control layer priority is generally disrupted according to the application requirements.
- The network and routing layer: Routing-disturbance attacks lead to DoS threats in multi-hop medical sensor environments. Generally, the routing attacks involve spoofing; altering routing paths or replaying packets; selective forwarding; sinkhole; warm-hole, *etc.*
- Transport layer: It controls end-to-end links, and suffers mainly from two popular types of attacks, namely, flooding attacks and de-synchronization attacks. Flooding attacks generally are used to drain the memory resources by sending the control signals. In de-synchronized attack, attacker may disturbs the established link between two legitimate two ends nodes (*i.e.*, body sensor and base station) by re-synchronizing their transmission. As a result, it disturb network communication, and network resources exhaustion [66,67].

DoS attacks may damage the wireless healthcare application network, and can lead to the loss of the patient’s life. Thus these (DoS) attacks are always harmful to the mission-critical applications, such as location tracking, ambulatory, home care monitoring, *etc.* For more detailed studies of denial-of-service attacks, readers may refer to [60,66,67].

### 3.2. Privacy Issues

As wireless healthcare applications are not limited to monitoring the patient’s physiological data, but they also include emergency management, healthcare data access, electronic health records, *etc.* Further, individuals share their data with physicians (in a doctor-patient relationship), insurance companies (for insurance protraction), and health-coaches (as sports team trainers) or with family (as relatives’ support for recovery). So there is value in addressing the privacy issues that are ethical from a social point of view. We adopt the privacy definition from National Committee for Vital and Health Statistics (NCVHS), which is consultative board of the United States Department of Health and Human Services. “Health information privacy is an individual’s right to control the acquisition, uses, or disclosures of his or her identifiable health data [68]”. To maintain privacy, patients should have the rights to determine which data should be collected, used or disclosed. Any unauthorized collection or leakage of patient data could harm the patient. For example, an unauthorized person may use the patient data (such as, patient identity) for their personal benefit, such as for medical fraud, fraudulent insurance claims, and sometimes this may even pose life-threatening risks [69]. As the medical data is very sensitive by the European Union Data Protection Directive [70], thus, there questions arise: who owns medical information, and how to control the access to medical data? Further, in wireless healthcare applications, huge amount of health and life-style data are gathered that need close attention to who controls it, what is gathered, who has rights to access it and where/how/whether that data is stored or not [71].

Meingast *et al.* [37] have raised similar questions regarding patient privacy: (i) Who can have permission to own the data; (ii) what type of medical data, how much, and where should the data be collected; (iii) who can have permission to inspect the medical data; and (iv) to whom should medical data be revealed to without the patient’s consent. In [38] the authors have discussed the privacy threats that raise questions about healthcare success: *Identity threat:* if a patient loses or share their identity that can pose significant financial, physical and emotional harm to an individual. An insider may use patient identity for their personal benefit, e.g., he/she misuse the identity to obtain reimbursement (insurance claims) or misuse the identity to obtain medical services [72,73]. *Access threats*: generally a patient is self involved in the access threats, if he/she fails to convey their consent properly. For example, in the absence of patient consent, an insider may damage the patient’s data and harm the patient for their personal reasons. In a patient medical record system, insiders may modify the medical records intentionally. For example, suppose an insider wrongly alters the patient’s medical data, such as, illness conditions, severe allergies, and specifically blood type, all of which pose life-threatening risks. For more details readers may refer to [38].

Ramli *et al.* have reviewed the privacy issues in a pervasive healthcare monitoring system [74]. The authors identified a few privacy issues in pervasive healthcare, such as, misuse of medical information, leakage of prescriptions, eavesdropping on medical data, and social implications for the patient. *Misuse of medical information*: The patient health data flows on a wireless channel, therefore, it is open to all the wireless threats, such as eavesdropping and snooping. Thus patient privacy could be breached if an unauthorized person captures the wireless data and misuses it [74]. *Leakage of prescriptions*: the authors pointed out that medical prescription can be a big source of privacy violations. For instance, to transfer or sell the prescription data from pharmacy/doctor to third parties, since the medical prescriptions contain detailed information about a patient, *i.e.*, name, id, diseases, *etc.* [74,75]. Thus leakage of prescription data becomes a privacy issue. *Eavesdropping on patient medical information*: patient medical information drifts on the wireless links, which are easily monitored. The monitoring system records patient data from communication channels and extracts the valuable patient information. Thus eavesdropping is very simple for an attacker, while the patient data is transmitting from the body area network to the caregiver device. Hence, the patient privacy breached. *Social implications for the patient*: Another privacy issue concerns the social implications, where a patient is not able to make decisions for their own privacy. For instance an older person (*i.e.*, age 65 and above), especially if he/she suffers from dementia (*i.e.*, dementia is a loss of mental skills that affects daily life [76]). Other authors have discussed difficulties in managing privacy settings and lack of support in designing privacy-sensitive applications; for more details readers may refer to [74].

This section has shown that security and privacy issues directly influence patient life and the healthcare system, if there is any loophole in the application security, so it is recommended to the forthcoming research that robust security should be considered from the beginning of the application design. Furthermore, as we have seen above the security and privacy issues could breach patient physiological information, so there is a need for stringent regulation and laws that can act to mandate rigorous standards for protecting patient sensitive information. The next section discusses such stringent laws for healthcare providers and related organizations.

## 4. Regulations and Laws

Medical security and privacy is an imperative requirement in healthcare organizations all over the world, so there are many different regulations and acts that affect healthcare providers. In fact the regulations and acts vary greatly from country to country. Here we discuss the American Health Insurance Portability and Accountability Act of 1996 (HIPAA) [39] and the Health Information Technology for Economic and Clinical Health Act (HITECH) [77].

➢ HIPAA regulates many different rules to be followed by doctors, hospitals, healthcare organization and other health related professionals. The Act requires comprehensive data security measures for data administration policies, data safeguards and supporting systems. According to the Act, healthcare providers are clearly subjected to strict civil and criminal penalties (*i.e.*, either fine of $250,000 or imprisonment for 10 years) for those who acquire or disclose the patient health information for money-making or malevolent harm. Furthermore medical providers must guarantee that their system, and those of their business associates, ensures [78] the following:
  ○ Security and confidentiality, *i.e.*, patient health information is secure, and is in a proper format.
  ○ Providing protection against any infringements of security, confidentiality and integrity, if they occur.
  ○ Providing protection against unauthorized access to or usage of the patient health information.
  Further HIPAA regulates some other areas:
  ○ Secure patient health records, particularly from those who do not need them.
  ○ Establish systems that need user identities (*i.e.*, both internal staff and consumers).
  ○ Limit the access to sensitive data and applications to authorized personnel (*i.e.*, role-based access control).
  ○ Ensure integrity of patient health information throughout its life-cycle within the system.
➢ The HITECH Act includes provisions to enlarge the use of information technology (IT) to store, capture, transmit, properly share and use health data. It introduces the new Act that states that those who manage patient health information (PHI) should notify the affected individuals if there is any breach that discloses their PHI [77].

Although the involvement of wireless technology and Internet access are providing a low-cost communication infrastructure that is suitable for home care monitoring, ambulatory monitoring, hospital and clinical monitoring, and so on, it should also be considered that in some special cases such as medical emergencies, or disaster medical management that there may a need to disclosure the patient information to many people involved in the rescue activities, so regulations should have some flexibility and users also have to compromise with their privacy to some extent. The next section discusses the existing security mechanisms that can provide security to wireless healthcare applications, and ensure patient privacy.

## 5. Existing Security Mechanisms

Security mechanisms are processes that are used to detect, prevent and recover from security attacks. Although there are significant security mechanisms for traditional networks (*i.e.*, wired and *ad hoc*) they are generally not directly applicable to resource constrained wireless medical sensor networks, so this sub-section discusses the issues concerning existing security mechanisms, as follows:

### 5.1. Cryptography

As wireless medical sensor networks deal with sensitive physiological information, strong cryptographic functions (*i.e.*, encryption, authentication, integrity, *etc.*) are paramount requirements for developing any secure healthcare application. These cryptographic functions provide patient privacy and security against many malicious attacks. Strong cryptography requires extensive computation and resources, therefore selecting appropriate cryptography are a challenging task for resource hungry medical sensor nodes that can provides maximum security whilst utilizing the minimum resources. Further, the selection of cryptography system depends on the computation and communication capability of the sensor nodes. Some argue that asymmetric crypto systems are often too expensive for medical sensors and symmetric crypto systems are not versatile enough [79]. However, applying the security mechanisms to resource constrained medical sensors should be selected based on the following considerations: *Energy:* how much energy is needed to perform the crypto functions. *Memory:* how much memory (*i.e.*, read only memory and random access memory) is needed for security mechanisms. *Execution-time:* how much time is required to execute the security mechanisms.

### 5.2. Key Management

Key management protocols are fundamental requirements to develop a secure application. These protocols are used to set up and distribute various kinds of cryptographic keys to nodes in the network. Generally, there are three types of key management protocols, namely, trusted server, key pre-distribution and self enforcing [40,80]. (i) *Trusted server* protocols rely on a trusted base station responsible for establishing the key agreement in the network. It is considered that the trusted server protocols are well suited to hierarchical networks in the presence of unlimited resource gateways. Although, trusted server based schemes provide stronger security to hierarchical networks, in a real-time environment, a trusted server could become a single point for the entire network failure; hence, they are not suitable for critical applications (e.g., healthcare) [40]. (ii) *Key pre-distribution* protocols are based on symmetric key cryptography, where secret keys are stored in the network before the network deployment. The key pre-distribution protocols are easy to implement, and offer relatively less computational complexity, making them more suitable for resource constrained sensor networks. (iii) *Self enforcing* protocols using a public-key infrastructure provide many advantages, such as, strong security, scalability, and memory efficiency. Earlier public key based solutions were thought to be too computationally expensive (*i.e.*, RSA [81] and Diffie-Hellman key exchange [82]) for wireless sensor networks. However, some researchers [83–85] have shown that Elliptic curve cryptographic based schemes are viable on resource constrained networks. In fact, in real-time implementation, the ECC based necessary cryptographic primitives (e.g., signature generation and verification) are still expensive in term of the time complexity.

### 5.3. Secure Routing

In home care or disaster scenarios sensor devices might require sending their data to other devices outside their immediate radio range [44]. Therefore, routing and message forwarding is a crucial service for end-to-end communication. So far, numerous of routing protocols have been proposed for sensor networks, but none of them have been designed with strong security as a goal [59,86]. Karlof-Wagner [61] discussed the fact that routing protocols suffer from many security vulnerabilities, such as an attacker might launch denial-of-service attacks on the routing protocol. An attacker could also inject malicious routing information into the network, resulting in inconsistencies in the routing. Further, most of current proposals are designed for static wireless sensor networks but mobility has not been taken under consideration, whereas healthcare applications require mobility supported routing protocols. In addition, designing secure routing protocols for mobile networks is a complex task and current WMSNs healthcare security requirements will make it more complex when they become real-time applications.

### 5.4. Resilience to Node Capture

Resilience against node capture is one of the most challenging problems in sensor networks. In real-time healthcare applications, the medical sensors are placed on a patient’s body, whereas, the environmental sensors are placed on hospital premises (e.g., ward room, operation room *etc.*) which may be easily accessible to attackers. Thus, an attacker might be able to capture a sensor node, get its cryptographic information and alter the sensor programming accordingly. Later, he/she can place the compromised node into the network, and that could endanger application success [87]. The current cryptographic functions (*i.e.*, node authentication and identification) may detect and defend against node compromised attacks to some degree, but these compromised node attacks cannot be detected instantly [87], which is a big issue for healthcare application. For example, consider the case of a false alarm. One possible solution to prevent this attack is to use tamper resistant hardware; however, tamper resistant hardware is not a cost effective solution.

### 5.5. Secure Localization

WMSNs facilitate mobility for patient’s comfort, therefore patient location estimations are needed for the success of healthcare applications. Since, medical sensors’ sense physiological data of an individual, they also need to report the patient’s location to a remote server. As a result, medical sensors have to be aware of patient location, *i.e.*, called localization. In [88] the authors discussed localization systems, which were divided into: distance/angle estimation, position computation and localization algorithms, and further, they discussed attacks on localization systems. In [86,87] the authors argue that mobility supported secure localization protocols still need to be explored.

### 5.6. Trust Management

Trust signifies the mutual association of any two trustworthy nodes (*i.e.*, sensor node and data aggregator node), that are sharing their information. In [89] trust is defined as “the degree to which a node should be trustworthy, secure, or reliable during any interaction with the node”. Wireless healthcare applications depend on distributed cooperation among the network nodes. The key aspect of healthcare applications is a trust evaluation on the behavior of a node (*i.e.*, data delivery and quality), so trust management systems are useful to detect the degree of trust of a node. Boukerche-Ren [89], evaluated the trust for mobile healthcare system. However, trust management must still be implemented in real-time healthcare application using WMSNs, to ensure a clearer picture of trustworthiness of the parties involved (*i.e.*, medical sensors, *etc.*).

### 5.7. Robustness to Communication Denial-of-Services

An attacker attempts to disrupt the network’s operation by broadcasting high-energy signals. If the broadcasting is powerful enough, then the entire network communication might be jammed. Other attacks are also possible, such as an adversary may delay communication by violating the medium access control protocol. Moreover, an adversary can transmit packets while a neighbor node is also transmitting. Raymond-Midkiff [67] has discussed the details of DoS attacks and their countermeasures at different layers of WSN routing, as shown in Table 2. Most of the DoS countermeasures are suitable for static wireless sensor networks, as shown in Table 3. Since, the WMSN healthcare applications are mobile in nature, as a result, secure DoS attack countermeasures still need further investigation for real-time healthcare application using WMSNs.

As we have seen in the above section there are tremendous robust security mechanisms but these mechanisms are not directly applicable to healthcare applications where resource constrained devices are used. Consequently, the security gap between the above securities measures are still needs to explore for healthcare applications.

## 6. Security and Privacy Requirements of Healthcare Applications

Based on the above application scenarios, security issues and regulatory laws, this section points out the paramount security and privacy requirements for healthcare applications using wireless medical sensor networks, as follows:

1. Data confidentiality: Patient health data are generally held under the legal and ethical obligations of confidentiality. These health data should be confidential and available only to the authorized doctors or other caregivers. Thus, it is important to keep the individual health information confidential, so that an adversary cannot eavesdrop on the patient’s information. Data eavesdropping may cause damage to the patient because the adversary can use the patient’s data for many illegal purposes and hence, the patient’s privacy is breached. Therefore, data confidentiality is an important requirement in healthcare applications using WMSNs.
2. Data authentication: Authentication services provide authorization, which is necessary for both medical and non-medical applications. In WMSN healthcare applications, authentication is a must for every medical sensor and the base-station to verify that the data were sent by a trusted sensor or not.
3. Strong user authentication: The major problem in a wireless healthcare environment is vulnerability of wireless messages to an unauthorized user, so it is highly desirable that strong user authentication should be considered, whereby each user must prove their authenticity before accessing any patient physiological information. Furthermore, strong user authentication, also known as two-factor authentication, provides greater security for healthcare applications using wireless medical sensor networks [78].
4. Data integrity: Data integrity services guarantee at the recipient end that the data has not been altered in transit by an adversary. Due to the broadcast nature of the sensor network, the patient’s information could be altered by an adversary; this could be very dangerous in the case of life-critical events. To verify the data integrity, one must have the ability to identify any data manipulation done by illegal parties. Thus, proper data integrity mechanisms ensure that the received data has not been altered by an adversary.
5. Key distribution: If two parties exchange information, they must share a session key and that key must be protected from others. A secure session key helps secure subsequent communication and safeguards data against various security attacks. Thus in order to preserve the patient’s privacy, an efficient key distribution scheme is a major requirement in wireless healthcare applications [90].
6. Access control: In healthcare application many users (such as doctors, nurses, pharmacists, insurance companies, lab staff, social workers, *etc.*) are always directly involved with the patient’s physiological data, so it is highly desirable that a role-based access control mechanism should be implemented in real-time healthcare applications that can restrict the access of the physiological information, as user’s roles. For example, the HL7 Standard Development Organization uses a role-based access control model [91].
7. Data availability: Availability ensures that services and information can be accessed at the time when they are required. Thus, medical sensor node availability ensures that the patient’s data are constantly available to the caregiver. If a sensor node is captured by an adversary, then its data availability will be lost, thus it is required to maintain always-on the operation of the healthcare applications in the case of loss of availability.
8. Data freshness: In healthcare applications, data confidentiality and integrity are not enough if data freshness is not considered. Data freshness implies that the patient physiological signs are fresh or resent; and thus an adversary has not replayed the old messages. There are two kinds of freshness: weak freshness, which gives partial message ordering but does not carry time-delay information; and strong freshness, gives a total order on a request-response pair and allows for delay estimation [92].
9. Secure localization: In healthcare applications, estimation of the patient’s location is very important. In real-time applications, a lack of smart patient tracking allows an attacker to send incorrect patient’s location by using false signals [92].
10. Forward and backward secrecy: In a real-time healthcare application, generally new medical sensors are deployed when old sensors fail, so it is important to consider forward and backward secrecy. In forward secrecy, a medical sensor cannot read future messages transmitted after it leaves the network, while in backward secrecy a sensor joining the network cannot read any previously transmitted messages [93].
11. Communication and computation cost: Since wireless medical sensors are resource constrained devices, and healthcare application’s functions also need room for executing their tasks, the security schemes must be efficient in terms of the communication and computational cost.
12. Patient permission: A patient’s permission is needed when a healthcare provider is disseminating his/her health records to another healthcare consultant. For example, medical researcher, insurance company, *etc.*

In addition, patient’s *anonymity* is also needed for healthcare applications because medical sensor networks are wireless in nature. Thus *anonymity* hides the source of a packet (*i.e.*, medical sensor data) during wireless communication. It is a service that can enable confidentiality. Further, a wireless healthcare application should enable minimum *survivability* in the presence of power loss, failures or attacks.

## 7. Related Works

This section discusses the recently published literature on secure healthcare monitoring using wireless sensor networks. In [9] Muhammed *et al.* proposed a biometric based distributed key management protocol, named BARI+, for wireless body area networks. The BARI+ architecture consists of a PS (personal server), MS (medical server), and WBAN (wireless body area network). In their scheme the WBAN is managed by four keys, namely, communication key, administrative key, basic key, and secret key that shared by sensor node and medical database/server. The BARI+ protocol is divided into three phases: (i) *initial deployment phase*: all initial keys are deployed in the PS, MS and WBAN in this phase; (ii) *re-keying phase*: in order to refresh the communication key, PS computes a value from the patient’s biometrics, encrypts it, and broadcasts it into the network; and (iii) *node addition phase*: if a new node is added then the MS informs the PS about new deployments by sending identities, basic keys and other relevant information of the new node to the PS. Further, the authors claim that their protocol facilitates many security services such as confidentiality, authentication, and security against replay attacks, forward secrecy, security against node compromise and security against routing attacks. Muhammed *et al.* simulated their scheme for Mica2 mote and they used RC5 block cipher for confidentiality and SHA-1 for hashing.

Waluyo *et al.* [32] proposed a lightweight middleware for personal wireless body area networks. The idea of the proposed middleware is to simplify and accelerate the development of wireless healthcare applications by using highly reusable codes. The middleware architecture has the following features: data acquisition, on-the-fly sensor reconfiguration, plug-and-play capabilities and resource management. In addition, it also provides security to protect critical sensor data from unauthorized parties. The authors choose SkipJack 64-bit lightweight block cipher cryptosystem for confidentiality, which consumes 622 bytes of ROM. For the performance evaluation three BSN accelerometers and one ECG were used as a prototype. The proposed middleware resides in a personal digital assistant (PDA).

Huang *et al.* [56] proposed secure access to a hierarchical sensor-based healthcare monitoring architecture. The healthcare architecture is composed of three network tiers (sensor, mobile, and back-end network) and has been demonstrated for three different pervasive healthcare applications (in-hospital, in-home, and nursing-home). In the *sensor network tier*, a wearable sensor system (WSS) using Bluetooth and integrated with biomedical sensors is used to monitor the vital signals of individuals. Wireless sensor motes [WSMs (*i.e.*, Mica2)] are placed within the building to collect the environmental parameters. WSS and WSM securely broadcast physiological information and environmental parameters to the upper layer. WSS uses an advance encryption standard (AES) based authentication (*i.e.*, CBC-MAC) and an encryption scheme, while the WSMs use a polynomial-based encryption scheme to establish secure point-to-point communication between two WSM motes. A public-key based key establishment protocol is used to establish the secure keys. In *the mobile computing network tier*, mobile computing devices (MCD) such as PDAs organized as in an *ad-hoc* network, route the data via multi-hops to the local station. MCD has the computational capabilities required to analyze the WSS and WSM data. A mobile-to-mobile text-based alarm message is used to show any real time abnormalities. Every MCD supports secure short message service (SMS) using cellular networks. Further, the authors used the ARAN routing protocol which is based on an *Ad Hoc* On-Demand Distance Vector (AODV) routing protocol for *ad-hoc* networks. The back-end tier is structured with a fixed station and server that provide application level services for lower tiers and process various sensing data. Although, the authors implemented the ARAN routing protocol in healthcare applications, their study did not show the different effects, e.g., energy consumption, memory requirements, *etc.* Therefore more analysis is needed to implement the secure routing protocols in the real-time healthcare applications. Furthermore, the authors claim that their scheme provides confidentiality, authentication and integrity.

Muraleedharan-Osadciw [60] proposed a secure health monitoring network against denial-of-service attacks using cognitive intelligence. They proposed energy-efficient cognitive routing protocol that copes with the Sybil and worm-hole attacks for healthcare applications.

Le *et al.* [79] proposed a MAACE protocol where an authorized professional can access the patient’s data. Their scheme provides mutual authentication and access control, which is based on elliptic curve cryptography (ECC). Furthermore, the authors claim their scheme can defend from real-time attacks, such as replay attacks, and denial-of-service attacks. The MAACE architecture is composed of three layers, namely, a sensor network layer (SN), a coordination network layer (CN), and a data access layer (DA). In this scheme, the SN transmits data to the CN (*i.e.*, PDA, laptop or cell phone), later, the data is forwarded to the DA for future records. Although, Le *et al.*’s protocol provides enough security, it is susceptible to information-leakage attacks, which could be dangerous for a patient’s privacy. As a result, the patient’s vital signs are exposed to unauthorized users, which is not acceptable for real-time healthcare applications.

Malasri *et al.* [83] implemented a secure wireless mote-base medical sensor network for health care applications. Their scheme has three main components: (a) A two tier scheme is used for data authentication based on the patient’s biometric and physiological data. (b) An ECC-based secure key exchange protocol is used to set up shared keys between the medical sensor and the base station. (c) A symmetric encryption/decryption algorithm is used for data confidentiality and integrity. Their security mechanism is as follows: each medical mote (Motive T-mote) is connected with a small fingerprint scanner. The patient’s identity is verified by the base station via the patient’s biometric signature. The query and patient data are encrypted using RC5 block cipher cryptosystem and data integrity is provided by a Hashed message authentication code (H-MAC). An elliptic curve cryptographic (ECC) based key exchange protocol is used between the medical sensor and the base station. Further, the scheme consumes 24.7 KB of ROM and 2.8 KB of RAM, and ECC operation takes 5.3 seconds for key exchange. Their scheme is secure against spoofing and physical compromise of motes, and provides confidentiality, authentication and integrity.

Boukerche-Ren [89] proposed a secure mobile healthcare system using a trust-based multicast scheme. A multicast strategy is used that employs trust to evaluate the behavior of each node. By doing so, only trustworthy nodes are permitted to participate in communications, while the misbehavior of malicious nodes identifies them and they are successfully prevented from communicating. The authors have brought forth a new trust evaluation theory whereby a node is only allowed to join the communication initiated by the source node when this new node is trustworthy for the source node.

Misic *et al.* [90] proposed two key distribution algorithms for enforcing patient privacy in healthcare WSNs through the use of key distribution algorithms. In the first algorithm, a central trusted security server is used to authenticate the participants that belong to the patient group and generate a session key. Four entities are involved in clinical system, namely, patient, clinician, nurse and central authority. In the second algorithm, an independent certificate is used for participant authentication, which is based on public key cryptography. The authentication is accomplished through the following steps: certificate generation; exchange of challenges; request of control coordinator (CTSS); base point distribution; confirmation of the reception of base point; and symmetric key generation. For detailed specifications the reader may refer to [90]. The authors proposed and analyzed two key distribution algorithms but they did not simulate or implement them in a real-time environment.

Haque *et al.* [94] proposed an efficient security scheme for patient monitoring systems using wireless sensor networks. Their scheme uses a public key based infrastructure, and is composed of three main components: patient (PT), healthcare service system (HSS), and secure base stations (SBSs). A pseudo-inverse matrix is used to derive a pair-wise shared key, and a bilateral key handshaking method is used to establish secure communication between HSS and SBS or PT and SBS. Because SBS has prior knowledge of the secret keys of PTs and HSSs, any node PT in the network can establish secure PT-to-SBS communication or *vice versa*. In addition, their scheme provides data confidentiality (*i.e.*, encryption and decryption).

Hu *et al.* [95] proposed a software and hardware based real-time cardiac patient healthcare monitoring system known as tele-cardiology sensor network (TSN). TSN is specially designed for the U.S. healthcare community, and performs real time healthcare data collection for elderly patients in large nursing homes. In TSN, a patient’s ECG signals are automatically collected and processed by small ECG sensors and transmitted wirelessly to an ECG server for further analysis. TSN is composed of a large number of wireless ECG communication units; each unit is called a mobile platform. In order to protect the patient privacy, TSN facilitates confidentiality (*i.e.*, only source and destination can recognize the medical data, who has knowledge of secret keys), and integrity (*i.e.*, no data is altered during the wireless communication). Further, to achieve the energy-efficiency cluster based routing is used. *Intra-cluster security*, skipjack block cipher cryptography algorithm used to protect patient physiological (*i.e.*, ECG data), and an *inter-cluster security* uses pre-distributed session keys. For multiple patients, cluster based routing used to reduce the patient-to-doctor routing overhead, and achieved efficiency. Their security scheme consumes 26 mJ (milli-joules) for data processing, 1,002 mJ for radio communication, and 11 mJ for memory accesses. For more details the reader may refer to [95].

Dagtas *et al*. [96] proposed another real time and secure architecture for health monitoring in smart homes using ZigBee technology. The proposed framework has the following features: (a) the ability to detect signals wirelessly within a body area sensor network (BSN); (b) low-power and reliable data transmission using ZigBee technology; (c) secure transmission of medical data over BSN; (d) efficient channel allocation over wireless networks, and (e) optimized analysis of data using an adaptive framework that maximizes the processing and computational capacity. A secure key management protocol used to establish secure session keys in body sensor networks because it provides cryptographic keys that facilitate security services, e.g., confidentiality, authentication, and data integrity. An authentication algorithm is used between the body sensors and the handheld device of the mobile patient. However, the authors provide security to physiological data, but they did not discuss which symmetric cryptosystem they have used, and did not analyze the energy efficiency for security services.

Kang *et al.* [97] proposed a wearable context-aware system for ubiquitous healthcare. The proposed context-aware system is composed of wearable sensor systems, wearable computers and communication modules. The wearable sensors are connected to wearable computers via ZigBee communication. The wearable sensor system has two types of sensor, *i.e.*, a watch type sensor and a chest belt type sensor. Wearable computers, e.g., PDAs, are used to collect the sensors’ data. ZigBee technology is used for communication between the wearable sensors and PDAs. A wireless local area network (LAN) with 802.11b (Wi-Fi) is used for communication between PDAs and healthcare service providers on the Internet.

Lin *et al* [98] proposed a strong privacy-preserving scheme against global eavesdropping for eHealth systems known as SAGE. The system achieves content oriented privacy and contextual privacy against a strong global adversary. The content oriented privacy applies if a malevolent user has the capability to disclose a patient’s data while data is being transmitted on wireless channels by observing and manipulating it. If not, then content oriented privacy is achieved. The contextual privacy stated that if a malicious user has the ability to link the data source (*i.e.*, patient) and the destination (*i.e.*, doctor/central server) of a message, then there is a contextual privacy breach. The functions of SAGE include system settings, patient registration, patient health information transmission and patient health information collection. The basic idea of SAGE is that a patient information database (PIDB) receives the personal health information (PHI) from patient’s body sensors (e.g., accelerometer, blood pressure, oxygen saturation, and temperature sensors); it broadcasts the PHI to all physicians. Then only the applicable physician has access to the patient’s PHI. In this system, all PHI information’s are stored in the PIDB at the eHealth center. To achieve access control, only registered patients can store their data and only legal physicians can retrieve patients’ data from the PIDB. Authors have proposed elliptic curve cryptography based privacy solution and demonstrate a formal proof for the proposed solution against strong eavesdropping. For more details refer to [98].

Kumar *et al.* [99] proposed a secure health monitoring (SHM) using medical wireless sensor networks. SHM provides security services such as confidentiality, authenticity, and integrity to the patient data at low computation and communication cost. The proposed scheme has the following components: (a) the ability to detect ECG signals wirelessly within the patient body, (b) low-power and reliable data transmission using Telos-B technology. In SHM, the confidentiality is achieved by PingPong-128 stream cipher cryptography, and authentication and integrity are achieved by PingPong-MAC, *i.e.*, message authentication code. Further, the proposed SHM consumes 1.1 KB RAM, 17.4 KB ROM and 19.2 ms CPU computation time.

Wu *et al.* [100] proposed an adaptive fault-tolerant communication scheme, named as AFTCS, for body sensor networks. AFTCS provide reliable data transmission for critical sensors by reserving channel bandwidth according to the amount of human physiological data, the external environment and the system itself.

As we have seen in this section, significant research has been conducted in order to secure healthcare applications using wireless medical sensor networks. It is obvious that extensive security and privacy research is needed in wireless healthcare application, which can fill the security gaps that we have discussed in the above sections.

## 8. Discussion

Wireless medical sensor networks make patients’ life more comfortable and provide viable solutions for healthcare applications such as vital sign monitoring, hospitals, home care, ambulatory care, clinical monitoring. The success of wireless healthcare absolutely depends on security as well. Security in healthcare application using wireless medical sensor networks is an emerging research topic and it is worth studying. This paper provides a fairly comprehensive study of security research in healthcare application using WMSNs.

In wireless healthcare, patient health data floats on wireless communication, so malevolent users must not be underestimated. Since wireless communication ranges are not confined, malevolent threats even pose more significant risks to the patient. For example a patient’s body data can be accessed in an unauthorized manner, can be modified, and consequently this can pose a life-threatening risk. Currently, appears that researchers have limited awareness of the patient risks. Most of the popular research projects acknowledge the issue of security, but they fail to implement strong security services that could preserve patient privacy (*cf.* Section 2), e.g., CodeBlue [7], Alarm-Net [13], UbiMon [14], MobiCare [16,53], STAIRE [55]. As result, a healthcare provider using [7,13,14,16,53,55] a system that permitted malevolent or others user to access the health data could be held liable for violating the Health Insurance Portability and Accountability Act (HIPAA) [39].

However, upcoming work is required in particular to consider the security and privacy issues for such applications where people life’s at high risk. To maintain strong security in a real-time healthcare application, security and privacy needs to be included from the starting point of application design, deployment, and implementation.

HIPAA regulated stringent rules for healthcare providers (*cf.* Section 4), *i.e.*, individuals’ vital signs are exposed to authorized professionals (*i.e.*, doctors, caregivers and nurses) [39,78]. A strong user authentication (*i.e.*, professional authentication) protocol at the application layer has not yet been addressed effectively in order to prevent illegal access to wireless medical sensor data, so user authentication is highly desirable in such wireless healthcare applications.

The aim of wireless healthcare is to let patient to survive when he/she is alone or wants to live an independent life, so mobility of the patient must be maintained, therefore there is need of such a security mechanism that can quickly adapt to dynamic topologies. However, little research deals with dynamic topologies, such as Alarm-Net, where the project is platform dependent. It could not support a secure handover mechanism to another network device, if a patient moves from a home network to a foreign network. So, future researchers or projects might pay attention to securing patient mobility while he/she is moving from network to network.

Researchers also have to carefully consider communication overhead, because any security protocols add computation and communication overhead. Since a high throughput is essential for health data, security protocols should be cost effective in terms of computation and communication costs.

Wireless healthcare applications need time for a synchronization protocol to compute end-to-end delay of packets for real-time analysis. Thus, the healthcare monitoring applications should be time synchronized that compute the end-to-end time delay between the medical sensors and the base-stations. More specifically, the time synchronization is more important for patient location tracking because patients’ body sensors need to do collaborative work such as sensing tasks, patient tracking, data routing and data aggregation. However, in healthcare application none of the aforementioned time synchronization schemes were designed with security in mind.

Furthermore, it is very clear that there are plenty of unsolved research needs special attention to explore security (recall Section 5) in healthcare application using WMSNs, as follows:

1. Public key cryptography: Recent studies have revealed that public key operation may be practical in medical sensors. But private key operations are still too expensive in term of time complexity, so the efficiency of private key operations still needs to be explored. Moreover, the authenticity of public key is highly desirable, e.g., suppose a body sensor receives a public key from a server, then there is the need to check the authenticity of the public key (*i.e.*, real or fake). In [56,93] the authors did not consider public key authentication. Since healthcare applications deal with sensitive patient data, in future, researchers should be mindful of the authenticity of public keys.
2. Symmetric key cryptography: Although, symmetric key cryptography is easy to implement and superior to asymmetric key cryptography (*i.e.*, public and private key) in terms of time complexity and energy cost, the demerit of symmetric key cryptography for medical sensor networks is that, it is not perfect for key distribution. Therefore, efficient and flexible key distribution protocols need to be designed for healthcare application using WMSNs.
3. Secure routing: Generally, most current secure routing protocols are designed for stationary networks, whereas medical sensor networks provides mobility to their patients and demands for mobility-supported multi-hop routing protocols [7,54,56,57]. Recalling the project described in [7], CodeBlue uses TinyADMR routing which is susceptible to routing loop attacks, grey-hole attacks, and Sybil attacks [49], so secure routing needs special attention for future wireless healthcare research.
4. Security and quality-of-service (QoS): Most healthcare studies consider security as an individual topic, whereas QoS [101–104] with security could become a tradeoff for healthcare applications using medical sensor networks, so security and QoS need to be evaluated jointly.

## 9. Conclusions

This survey discussed the security and privacy issues in healthcare applications using medical sensor networks. It has been shown that a well-planned security mechanism must be designed for the successful deployment of such a wireless application. In this respect, we have found many important challenges in implementing a secure healthcare monitoring system using medical sensors, which reflects the fact that if a technology is safe, then people will trust it. Otherwise, its use will not be practical, and could even endanger the patient’s life. Consequently, many security and privacy issues in healthcare applications using wireless medical sensor networks still need to be explored and we hope that this survey will motivate future researchers to come up with more robust security mechanisms for real-time healthcare applications.

## Figures and Tables

**Table 1.** Difference between generic WSN and WMSN.

**Generic WSNs**	**WMSNs**
Automatic and standalone	Human involvement
Scalability (*i.e.*, large scale)	Scalability (*i.e.*, small scale)
Fixed or distributed deployment	Mobility
Reliability (data rate depend on applications)	Reliability (high data rate)

**Table 2.** Denial-of-service attacks at each network layers.

**Layers**	**Attacks**
**Physical layer**	Jamming, Node tampering
**Link layer/medium access control**	Collision, exhaustion, and unfairness
**Network and routing layer**	Neglect and greed, homing, misdirection, spoofing, replaying, routing-control traffic or clustering messages
**Transport layer**	Flooding and De-synchronization
**Application layer**	Overwhelming sensors, reprogramming attack

**Table 3.** Denial-of-Service attacks and countermeasures at each network layer.

**Network Layer**	**Attacks**	**Countermeasures**
**Physical layer**	Jamming	Detect and sleep, route around jammed areas
	Node tampering	Temper-proof boxing
**Link layer/medium access control**	Collision, unfairness and	Authentication and anti-replay protection
	Denial of sleep	Authentication and anti-replay, detect and sleep, broadcast attack protection
**Network and routing layer**	Neglect and greed, misdirection, spoofing, replaying, routing-control traffic or clustering	Authentication and anti-replay protection, Secure cluster formation
	Homing	Header encryption and dummy packets
	Hello floods	Pair-wise authentication, geographic routing
**Transport layer**	Flooding	SYN cookies
	De-synchronization	Packet authentication
**Application layer**	Overwhelming sensors	Sensor tuning, data aggregation
	Reprogramming attack	Authentication and anti-replay protection Authentication streams
	Path-based DoS	Authentication and anti-replay protection
